# Proteomic Characterization of Antibiotic Resistance in *Listeria* and Production of Antimicrobial and Virulence Factors

**DOI:** 10.3390/ijms22158141

**Published:** 2021-07-29

**Authors:** Ana G. Abril, Mónica Carrera, Karola Böhme, Jorge Barros-Velázquez, Pilar Calo-Mata, Angeles Sánchez-Pérez, Tomás G. Villa

**Affiliations:** 1 Departamento de Microbiología y Parasitología, Facultad de Farmacia, Campus Sur 15782, Universidad de Santiago de Compostela, 15782 Santiago de Compostela, Spain; anagonzalezabril@hotmail.com; 2 Marine Research Institute (IIM), Spanish National Research Council (CSIC), Eduardo Cabello 6, 36208 Vigo, Spain; 3 Agroalimentary Technological Center of Lugo, Montirón 154, 27002 Lugo, Spain; KarolaBoehme@gmx.de; 4 Departamento de Química Analítica, Nutrición y Bromatología, Área de Tecnología de los Alimentos, Facultad de Veterinaria, Campus Lugo, Universidad de Santiago de Compostela, 27002 Santiago de Compostela, Spain; jorge.barros@usc.es (J.B.-V.); p.calo.mata@usc.es (P.C.-M.); 5 Sydney School of Veterinary Science, Faculty of Science, University of Sydney, Sydney, NSW 2006, Australia; angelines2085@icloud.com

**Keywords:** LC–ESI–MS/MS, proteomics, mass spectrometry, antibiotic resistance peptides, antibiotic production, virulence factors, *Listeria* spp.

## Abstract

Some *Listeria* species are important human and animal pathogens that can be found in contaminated food and produce a variety of virulence factors involved in their pathogenicity. *Listeria* strains exhibiting multidrug resistance are known to be progressively increasing and that is why continuous monitoring is needed. Effective therapy against pathogenic *Listeria* requires identification of the bacterial strain involved, as well as determining its virulence factors, such as antibiotic resistance and sensitivity. The present study describes the use of liquid chromatography–electrospray ionization tandem mass spectrometry (LC–ESI–MS/MS) to do a global shotgun proteomics characterization for pathogenic *Listeria* species. This method allowed the identification of a total of 2990 non-redundant peptides, representing 2727 proteins. Furthermore, 395 of the peptides correspond to proteins that play a direct role in *Listeria* pathogenicity; they were identified as virulence factors, toxins and anti-toxins, or associated with either antibiotics (involved in antibiotic-related compounds production or resistance) or resistance to toxic substances. The proteomic repository obtained here can be the base for further research into pathogenic *Listeria* species and facilitate the development of novel therapeutics for these pathogens.

## 1. Introduction

*Listeria* are Gram-positive facultative anaerobic bacteria that form small rods with low GC content; the species do not form spores or capsules and are motile at temperatures between 10 °C and 25 °C [1]. *Listeria* colonize a wide range of different environments, and species of this genus can be isolated from foodstuffs such as milk, poultry, and meat. Species such as *Listeria monocytogenes* and *Listeria ivanovii* can be spread and transmitted through contaminated food. *L. monocytogenes* represents the main human and animal pathogen that causes listeriosis [2]; *L*. *ivanovii* was also described, although to a lesser extent, to cause stillbirths or premature births in ruminants [3], while two additional species (*Listeria innocua or L. grayi*), that were originally considered nonpathogenic, are reported to also infect humans [4]. *Listeria* contamination is common in the food industry, as these pathogens can colonize both the equipment and the environment, forming biofilms that can survive for months or even years [5]. Fortunately, listeriosis incidence is low in the general population, as the syndrome presents with a high mortality rate (20–30%) in susceptible individuals with severe symptoms [6]. Four *L. monocytogenes* serovars—13, 1/2a, 1/2b, and 4b—are known to cause listeriosis, with 4b as the most frequent serovar found in humans [6].

*Listeria* species are currently still sensitive to a variety of antibiotics, such as penicillin, fluoroquinolones, macrolides, and vancomycin [4]. Antibiotic sensitivity has been mainly studied on *L. monocytogenes*, and the species is susceptible to a variety of antimicrobials, with the exception of cephalosporins and sulfonamides. The treatment of choice for listeriosis is currently either ampicillin or penicillin combined with aminoglycosides, with trimethoprim–sulfamethoxazole as an alternative. Recent studies have stablished that resistance to single antibiotics is more common than multiple resistance; tetracycline resistance is the most frequent, although some bacterial strains are resistant to other antibiotics [2,6,7]. As is generally the case for bacteria, the prevalence of antibiotic resistance, including multiple antibiotic resistances, within the genus *Listeria* spp. is currently increasing. A recent study demonstrated that a variety of *Listeria* species (i.e., *L. murrayi*, *L. welshimeri* or *L. gravi*) are sensitive to semisynthetic penicillins and vancomycin, but resistant to nalidixic acid [4]. The increase in antimicrobial resistance in bacteria present in food has been explained as follows: while antibiotic-resistant bacteria present in foods are initially saprophytic or commensal, their resistance genes are sporadically transferred to other foodborne microorganisms, thus contributing to worsen this problem. It is therefore currently urgent to develop an in-depth knowledge of how antibiotic resistance is disseminated among bacteria, in order to control this major threat to human and animal health and welfare [2].

Infection with pathogenic *Listeria* species involves at least eight known virulence factors. Bacterial invasion and breach of the gut epithelial barrier, after ingestion of contaminated food, is mediated by the bacterial surface proteins known as internalines (InlA and InlB). Escape from the host phagocytic vacuole is mediated by secreted bacterial proteins, such as PlcA, pore-forming toxin listeriolysin O (LLO), phosphatidylcholine-specific phospholipase, and phosphatidylinositol-specific phospholipase C. Intracellular actin-based motility (ActA) and cell-to-cell spread (without actual exit to the interstitial liquid) is accomplished by Hly and PlcB. All these genes are clustered into two pathogenicity islands, and the expression of the main virulence gene is controlled by transcription of the regulator PrfA, which is absent in *L. innocua* [3,6,8,9,10,11]. An additional 47 genes, scattered throughout the bacterial genome, have been identified to affect virulence [12].

*Listeria* detection and identification has usually focused on colony morphology, sugar fermentation and hemolytic properties; however, the methodology involved is time consuming and not appropriate to test food with a short shelf life. The food industry requires fast and unequivocal methods to quickly detect and identify pathogens present in foodstuffs. Although a number of procedures, such as molecular-based techniques (DNA hybridization, polymerase chain reaction (PCR), and microarray), enzyme-linked immuno-sorbent assay (ELISA), and flow cytometry, have been recently developed [13], these techniques need long enrichment time, expensive chemicals, and specialized equipment. Other methods developed for *Listeria* spp. detection and characterization include biosensors [14] and target proteomic methods [15,16,17]. In addition, matrix-assisted laser desorption/ionization time-of-flight mass spectrometry (MALDI-TOF MS) [15,16] and liquid chromatography coupled to tandem mass spectrometry (LC–MS/MS) against a spectral library construction, were used to stablish genotype–proteotype–phenotype relationships among the bacterial strains [17]. Another approach involved the development of shotgun proteomics protocols for the determination of virulence factors, such as adhesins, in *L. monocytogenes*, as well as the identification of relevant transport and metabolic pathways using LC–ESI–MS/MS [18,19,20]. Additional studies involve the use of LC–ESI–MS/MS to research the proteomics of antibiotic resistance and the production of antimicrobials and other virulence factors, in order to correctly identify the different streptococcal species associated to mastitis [21].

The characterization of virulence factors, pathogenicity, strain variation, and pathogenic serotypes is an essential part of epidemiologic investigations and food safety monitoring. The objectives of this study were to identify bacterial peptides corresponding to specific virulence factors, such as the production of antimicrobials, toxins and antimicrobial resistance, using advanced shotgun proteomic methods, to determine the diversity of the *Listeria* pathogens isolated from food.

## 2. Results

### 2.1. Listeria spp. Proteomics Data Repository

Nine different *Listeria* strains were studied (Table 1). Bacterial peptides were prepared by treating the protein mixtures with trypsin and analyzed by LC–ESI–MS/MS, as described previously [21,22,23]. A total of 2990 non-redundant peptides were identified which corresponded to 2727 annotated proteins from *Listeria* (see complete non-redundant dataset in Excel Supplemental Data). Accordingly, the virulence factors were identified by comparison with both the “Virulence Factors of Pathogenic Bacteria Database” (VFDB; http://www.mgc.ac.cn/VFs/, accessed on 26 July 2020), and with previously reported data [1,6,10,24,25].

In the group of non-redundant peptides, 395 were identified unequivocally as virulence factors, and included proteins such as the internalins involved in eukaryotic cell colonization and immune evasion, and which are specific for *Listeria*. Additional polypeptides (toxins and antitoxins), together with polypeptides so far involved in antibiotic resistance, were also found. The 395 virulent factors identified for listerial species are displayed in Tables S1–S6 in Supplemental Data and organized in groups according to the main role they play; this includes toxins, antibiotic resistance peptides, and other tolerance proteins involved in resistance to toxic substances, colonization and immune evasion factors, antimicrobial compounds, ABC and other transporters associated with virulence factors, and alternative virulence factors. In addition, the main antibiotic resistance proteins, antimicrobial related proteins, and other virulence factors are summarized in Table 2.

Two *Listeria* strains, Li4 and Li2 (with 73 and 116 peptides, respectively), *L. monocyogenes* and *Listeria seeligeri*, respectively, contained the highest number of peptides related to virulence. Taken together, all these results it suggests that the two species are probably the most pathogenic ones, as compared other species. Li2 strain, from *L. seeligeri*, was described as an avirulent Risk group 1 bacterium by Rocourt and Grimont (1983), it was also identified as non-pathogenic for holoxenic pathogen-free mice (50% lethal dose, more than 10′ colony-forming units per mL) [26]. In addition, strains Li1 (*Listeria welshimeri*) and Li3 (*Listeria innocua*) are also classified as risk Group 1; they are represented with 17 and 55 peptides related to virulence factors, respectively. The remaining strains analyzed belong to either risk group 1 or to different serotypes and are represented in Table 1 with a number of peptides related to virulence factor proteins that ranges from 15 to 37.

### 2.2. Proteins Involved in Bacterial Resistance to Antibiotics and Other Toxic Substances

In the present study 82 peptides, present in *Listeria* strains and involved in resistance to either antibiotics or toxic substances, were identified (Table S1). Forty-nine of the proteins were associated with antibiotic resistance, whereas the remaining 33 related to other tolerances.

Five peptides were characterized as belonging to the beta-lactamase family, while three peptides corresponded to a MarR family of transcriptional regulators, and one to a M56 peptidase family. An additional set of 15 peptides were characterized as penicillin-binding proteins, these exhibiting high affinity for the antibiotic [27]. MarR acts as a regulator for proteins involved in resistance against several antibiotics [28], while the M56 family protein is a metallopeptidase involved in beta-lactamase transduction [29].

Two peptides were characterized as aminoglycoside N(3)-acetyltransferases [30]. This enzyme can provide resistance against a variety of antibiotics, such as gentamicin, kanamycin, tobramycin, neomycin, and apramycin, which contain 2-deoxystreptamine rings; these rings acting as acceptors for the acetyltransferase activity. Two peptides were identified as part of proteins belonging to the family of fosfomycin resistance thiol transferases FosX/FosE/FosI and the tetronasin and bleomycin resistance, respectively. FosX proteins are Mn(II)-dependent fosfomycin-specific epoxide hydrolase and inhibit fosfomycin by hydration [31]. Four additional peptides were found to correspond to the GNAT family of acetyltransferases which confer antibiotic resistance by catalyzing the acetylation of amino groups in aminoglycoside antibiotics [32]. Two PhzF family phenazine biosynthesis protein were also identified by analysis of their peptides, and as it is known many phenazines exhibit broad-spectrum antibiotic activity against a variety of organisms, such as bacteria, parasites and fungi [33]. Ten peptides belonged to the TetR family of regulators (TFRs); the TetR proteins play a role in the regulation of genes encoding small-molecule exporters and antibiotic resistance. However, they also contribute to both antibiotic and quorum sensing production [34]. A peptide of a VanZ family protein O was also identified; these proteins confer resistance to teicoplanin, a glycopeptide antibiotic [35]. The last of the antibiotic resistance peptides presented in Table S1 is YdeI, a member of the YjbR/CyaY-like superfamily; this protein is located in the bacterial periplasm and interacts with OmpD/NmpC, playing an important role in antimicrobial peptide resistance [36]. The peptides associated with antibiotic resistance were detected in eight out of the nine *Listeria* strains studied (Li1, Li4, Li5, Li2, Li6, Li3, Li7, and Li8).

Bacterial tolerance refers to the ability of bacterial populations to survive treatment with antimicrobial agents, without developing resistance; many of the mechanisms governing bacterial tolerance directly influence the virulence of the strain. This study revealed peptides with a role in bacteria resistance and tolerance related proteins. One peptide interestingly related to an AIPR protein which is involved in resistance to abortive phage infection, and often found in restriction modification system operons [37]. Three peptides were identified as TelA, a protein belonging to the toxic anionic resistance family; TelA confers tellurite resistance [38]. Two further peptides corresponded to a Cass 2 protein and a copper resistance protein, respectively. Cass2 is an integron-associated protein that binds cationic drug compounds with sub-micromolar affinity [39]; while the copper resistance protein C (CopC) provides resistance to the metal by binding one atom of copper per protein molecule [40]. Four peptides were related to mercury resistance MerR proteins; these are metal ion sensing regulators that create a mercury-resistant phenotype via transcription of several Mer genes [41]. A peptide of an organic hydroperoxide resistance (Ohr) protein was also identified, this protein is involved in fatty acid peroxidation and the peroxynitrite bacterial response [42]. Two additional peptides represented a two-component sensor histidine kinase response to antimicrobials and a SugE protein, respectively; SugE is a small multidrug resistance protein [43]. Another peptide was identified as belonging to the quinolone efflux pump NorB [44]; efflux pumps play a role in the resistance of *L. monocytogenes* strains to antibiotics such as fluoroquinolones [14].

Additional peptides corresponded to proteins involved in other important bacterial tolerances, such as thermotolerance, osmotolerance, and stress tolerance. In addition to the major virulence factors involved in the pathogenicity of *Listeria*, a number of polypeptides, such as stress proteins, are also important, as they facilitate bacterial persistence in the environment as well as in the infectious process, and as matter of fact a number of publications indicate that both acid and oxidative stress proteins are produced following *Listeria* infection of human epithelial cells [45]. Accordingly, this study identified peptides belonging to heat shock proteins, such as the molecular chaperone Hsp70, Hsp20/alpha crystallin family of proteins and polypeptides such as DnaJ and ClpB, that are also chaperones involved in thermotolerance phenotypes. Both heat shock proteins (HSPs) and molecular chaperones DnaK and GroESL are induced during listerial infection of human epithelial cells [45,46]. In the case of bacterial heat damage, the heat-shock proteins DnaK, DnaJ and GrpE jointly induce ClpB synthesis; ClpB plays a role in thermotolerance, allowing *L. monocytogenes* to survive under increased, putative lethal, temperatures [45]. A total of three peptides were identified as general stress proteins, including a phosphoserine phosphatase RsbU. This protein dephosphorylates RsbV in response to environmental stress, acting as a general stress regulator of Gram-positive organisms [47]. Moreover, one of the peptides analyzed corresponded to proteins peroxiredoxin OsmC required for osmotic tolerance, an osmotically inducible protein [48]. One of the peptides constituted phenolic acid stress response proteins was also identified, PadR, an environmental sensor that acts as repressor of padA gene expression in the phenolic acid stress response [49]. Finally, all of the bacterial strains included in the present study contained two peptides corresponding to universal stress proteins.

Many bacterial transporters, such as the ABC transporter, play a role in either antibiotic and other resistances or tolerances; transporter proteins are described in Section 2.6.

### 2.3. Antibacterial Compounds and Proteins Involved in Antibacterial Production

This study identified two antibacterial proteins, from all the bacterial strains analyzed, which are present in the proteomic repository for *Listeria* spp. (Table S2); one of the proteins is involved in antibiotic production (antibiotic biosynthesis monooxygenase) found in strains L9 of *L. monocytogenes*, while the second one is a bacteriocin that belongs to the Lactococcin 972 family (found in strain L4 of *L. seeligeri*). A recent study demonstrated that *L.monocytogenes* strain EGD-e contains the *lmo2776* gene in its genome; *lmo2776* is homologous to the lactococcin 972, that produces a protein (Lcn972) that is secreted by *Lactococcus lactis*, *Streptococcus iniae*, *Streptococcus pneumoniae,* and *Staphylococcus aureus*; interestingly, the gene is absent in the non-pathogenic species *Listeria innocua* [50].

### 2.4. Proteins Involved in Bacterial Toxicity

The present study identified, by LC–ESI-MS/MS, 12 peptides involved in bacterial toxicity and recorded them in the proteomic repository for the bacteria (Table S3). The peptides correspond to proteins toxin Zeta, Type II toxin-antitoxin system, LXG domain-containing protein, and HicA toxin. Toxin zeta is a member of the epsilon/zeta TA family, described to stabilize resistance plasmids in major human pathogens, and regulated by the repressor protein omega; the toxin is released from epsilon by continuous antitoxin degradation through AAA+ proteases and causes cell death [51]. Members of the HicA toxin family contain a double-stranded RNA-binding domain, that functions as a translation-independent mRNA interferase; while HicB is the antitoxin which inhibits their mRNA interferase activity [52,53]. RelE/ParE family toxin is a member of the Type II toxin-antitoxin system that was also identified in this study. RelE toxins are mRNA interferases, while ParE toxins inhibit gyrase activity [54]. One of the peptides characterized corresponds to a Type II toxin–antitoxin system PemK/MazF family toxin; this family of proteins contains a toxin and an antitoxin gene pair as part of a post-segregation killing system, where gene loss results in the toxin attacking the cell. MazE, is the antitoxin that inhibits toxin MazF and, under stress conditions, mazEF transcription is reduced leading to the degradation of MazE, thus inhibiting cell division and resulting in cell death [55]. Seven peptides that belong to a LXG domain-containing protein, were identified in the *Listeria* strains (Li4, Li2, Li3, and Li7); this domain is present is the N-terminal region of a group of polymorphic toxin proteins.

### 2.5. Proteins Involved in Host Colonization and Immune Evasion

A total of 131 peptides were identified as belonging to proteins that play a role in *Listeria* colonization and immune evasion (Table S4).

These proteins are important for bacterial internalization into the mammalian host intestinal epithelial, thus facilitating listerial infection and propagation. These peptides represent proteins, such as internalin A (InlA) and internalin B (InlB), that facilitate bacterial entry into the host vacuole [11,56]. Additional bacterial surface proteins, such as adhesins, engage host receptors and also enable bacterial internalization [57]. This study identified so far fourteen peptides corresponding to internalins, five adhesin peptides, and six peptides representing Cna proteins. The adhesins identified included a polysaccharide intercellular polypeptide; this protein is encoded within the intercellular adhesin (*ica*) locus, involved in biofilm formation [58]. Cna is a collagen-binding MSCRAMM (Microbial Surface Component Recognizing Adhesive Matrix Molecules). To complete its infection cycle, *L. monocitogenes* must escape the host phagosome and reach the cytoplasm, and this role is facilitated by the competence (Com) system proteins [59].

Two peptides obtained from two of the *Listeria* strains analyzed were identified as ComGA (*L. welshmeri*) and ComEC in *L. monocytogenes* Li6 strain, respectively, being both proteins part of the Com system. Furthermore, one additional peptide was characterized as a phosphatidylinositol-specific phospholipase C (PI-PLC), which is used by *L. monocytogenes* uses the PI-PLC to destroy the membrane of the host vacuole and concomitantly releasing the bacterial cells into the cytoplasm [11]. Additional peptides included the chemotaxis protein CheA, which was present only in *L. monocitogenes* Li4 strain. As it is known, this protein facilitates bacterial intracellular movement, as it belongs to the Che group of proteins involved in flagellar rotation [56]. A total of 39 peptides were identified as peptidases, including members of the P60, M23, M22, M42, and M75 peptidase families, invasion associated secreted endopeptidase and viral enhancing peptidases. Particularly, the 60-kDa extracellular protein (p60) is a member of the P60 family that is encoded by the *iap* gene and participates in the host invasion process [60]. Peptidase C60 is also a sortase, a protein that plays a role in pili assembly by attaching secreted proteins to the cell wall; this peptidase is also involved in the infection process, rendering a good putative antibacterial target [61]. Two of the *Listeria* peptides were part of an autoinducer (AI-2) transporter (YdgG), that controls the transport of AI-2, the quorum sensing signal, and, consequently, represses biofilm formation [62].

A peptide of a peptidoglycan O-acetyltransferase was also identified; This protein is involved in avoiding eukaryotic lysozyme recognition by a versatile mechanism consisting on peptidoglycan acetylation and deacetylation [63]. Moreover, nine peptides belonging to the CLp ATase family were detected; CLp proteins are formed by a CLp ATase and a peptidase, the latter hydrolyzes the proteins controlling the modulation of virulence factors, such as biofilm formation [64,65].

Although *Listeria* species, as mentioned above, are described as non-capsulated, Smith and Metzger reported in 1962 that some strains of *L. monocytogenes* were able to form such a polysaccharide structure that can be related to virulence [66], although under certain circumstances, an unambiguous coat can be added to the bacterial wall which can be misidentified as a capsule [67]. This study characterized three peptides as related to the capsular exopolysaccharide protein family, and capsule biosynthesis was determined for tree of the strains analyzed (Li4, Li2, and Li9; *L. monocytogenes, L. seelingeri*, and *L.ivanovi*, respectively). In addition, three N-acetylmuramoyl-L-alanine amidase peptides and three D-alanyl-D-alanine carboxypeptidase peptides were identified also in this study, and the enzyme has been recorded as playing a role in virulence [68], N-acetylmuramoyl-L-alanine amidases are autolysins involved in bacterial adherence to eukaryotic cells. Some reports indicate that *Listeria* N-acetylmuramoyl-L-alanine amidases originate from phage endolysins [69,70]. Additional lysis proteins identified include muramidases and LytC, which are the main enzymes responsible for bacterial cell lysis during active growth [71]. Six listerial peptides contained the LysM domain.; this is a protein module, originally found in enzymes that degrade bacterial cell walls, present in many bacterial proteins involved in pathogenesis [72].

Transcriptional regulators, controlling virulence factors, were also identified for *Listeria* species; they include three peptides identified as LysR, five peptides corresponding to LytR and two peptides representing the LytTR transcriptional regulators. LysR contributes to *L. monocytogenes* virulence by controlling multiple pathways that include cationic antimicrobial peptide (CAMP) resistance, fructose and mannose metabolism, and beta-lactam resistance [24]. LytR and LytTR proteins regulate additional virulence factors, such as extracellular polysaccharides, toxins and bacteriocins, the polypeptides bind specific DNA sequences and act as transcriptional activators [8,73].

Two superoxide dismutase enzymes (SOD) peptides were also identified in the *Listeria* strains. SOD is a metalloenzyme that catalyzes superoxide radicals produced by host macrophages and neutrophils thus helping to fight the bacterial infection; in this sense the enzyme would facilitate the *Listeria* infection [74]. An additional group of four peptides corresponded to a Mga protein, another DNA-binding protein that regulates the expression of virulence genes; they included the M protein family of polypeptides (emm, mrp, and enn), C5a peptidase (scpA), and collagen-like protein 1 (scl1), that plays an important role in colonization and immune evasion [75]. Five of the peptides identified belonged to the well characterized type VI and type VII secretion systems and included an EssA peptide and an EssB peptide, both belonging to the Type VII system (ESS). This system facilitates the secretion of extracellular proteins across the cytoplasmic membrane and is involved in host infection [76]. A VirB/VirD4 component was also identified, this is a component of the type IV secretion system (T4SS) that is essential for both establishing intra- erythrocytic infection, as well as translocation of effector molecules into the host cells, thus causing a variety of effects [77].

The present study also identified one ESAT-6 peptide; which is a protein that plays an important role in virulence and immune evasion in *Mycobacterium tuberculosis*, with polypeptide homologues present in other Gram-positive bacterial species [78]. Moreover, one peptide was found to correspond to an insulinase; these are proteases that contains the M16 domain (metallopeptidases) and specifically cleave small proteins and peptides during bacterial invasion [79].

The most abundant glycopolymers in *Listeria* cell walls are teichoic acids (TAs) [80]. In addition, lipoteichoic acid (LTA) was reported to be involved in a wide range of inflammatory diseases; LTA is recognized by the eukaryotic Toll-like receptor 2 (TLR2), which triggers the innate immune responses [81]. Five TAs, LTA and related biosynthesis proteins were identified among the peptides obtained from *Listeria* strains. GtcA is protein that plays a role in teichoic acid glycosylation, that appears to be serotype-specific in some *L. innocua* and *L. monocytogenes* strains [82,83,84]. Another peptide was identified as protein DltD, a polypeptide involved in D-alanyl-lipoteichoic acid biosynthesis [81].

Finally, three *Listeria* peptides were identified as the transcriptional regulator Rgg, also called RopB. Rgg modulates the transcription in Gram-positive bacteria and is also required for secretion of several virulence-associated proteins. Additional, yet uncharacterized, Rgg-like proteins are encoded by the genomes of *Streptococcus* spp. and *L. monocytogenes* [85].

### 2.6. Transporters Associated to Virulence Factors

Several ABC-type transporters are involved in virulence and play an important role in the bacterial propagation during infection [86,87]. Putative ABC transporters representing virulence factors were identified in 113 of the *Listeria* peptides obtained, in addition to 12 peptides corresponding to a variety of transporters that facilitate bacterial virulence strategies (Table S5).

It is well established that bacteria, and in particular pathogenic strains, exhibit complex strategies in order to survive nutrient deprivation and in order to continue to growth, they respond to hardship conditions by releasing a variety of stress proteins and by immune evasion mechanisms. The LC–ESI-MS/MS analyses carried out here identified several peptides corresponding to proteins that are required for the uptake of metals, such as zinc and ferrous ion. Furthermore, some of peptides identified as ABC transporters are involved in the transport of fluoroquinolones, siderophores [88], oligopeptides ad peptides, amino acids, glycine/betaine [86], macrolide, spermidine, putrescine, bacitracin, petrobactin and other antibiotics, pheromones, and multidrug transporters. Multidrug ABC efflux transporters extrude antibiotics out of the bacterial cells thus allowing pathogenic bacteria to resist antimicrobial treatment [89]. The macrolide ABC transporters protect bacteria from macrolide drug treatment and are involved in both colistin and bacitracin resistance [90].

Additional, non-ABC, transporters related to virulence were also identified among the peptides analyzes. These peptides belong to a variety of transporters including cadmium, zinc and cobalt, putrescine, arsenical, multidrug MFS Mepa and SMR transporters, as well as chloramphenicol/florfenicol drug efflux MFS transporters. MFS (Major Facilitator Superfamily) is one of the largest groups of solute transporters [91], while the SMR (Small Multidrug Resistance) transporter family confers bacterial resistance to a variety of toxic elements, that are eliminated from the cells [92]. On the other hand, MepA is a member of the Multi Antimicrobial Extrusion (MATE) family, which acts as a drug/sodium antiporter through H^+^ or Na^+^ exchange. These proteins mediate resistance to a range of cationic drugs, such as fluroquinolones, aminoglycosides, norfloxacin, and ethidium bromide [93]. Finally, an additional multiple drug transporter which acts like an efflux pump across the cell envelope in bacteria is the resistance-nodulation-cell division (RND) protein family [94].

### 2.7. Other Bacterial Virulence Factors

The presence of mobile genetic elements is considered as perhaps the major mechanism of obtaining antibiotic resistances in *L. monocytogenes* [7,95]. These mobile elements may be either plasmids or viral DNA fragments, and provide a wide range of genes that encode proteins involved in antibiotic resistance and virulence determinants, as well as additional polypeptides playing a role in a variety of metabolic pathways [11,96]. Many peptides corresponding to proteins involved in the acquisition of these mobile elements were identified in the current analysis, including recombinases, integrases, and a phage infection protein (YhgE); additional peptides represented specific plasmid proteins, such as plasmid mobilization relaxosome protein MobC and transposases corresponding to different transposons, all of them related to a possible mechanism(s) of horizontal gene transfer among bacteria, were also found (Table S6). Four of the peptides characterized (Table S6) are part of the Tn3 transposase; this family of transposases can carry a variety of transient genes conferring antibiotic or heavy metal resistances, virulence and toxin–antitoxin (TA) system often associated with plasmid maintenance [30]. A peptide corresponding to a mutator family protein was also identified (Table S6); this polypeptide belongs to the class II DNA transposable elements (TEs) family that can exchange ectopic genomic sequences, leading to the formation of new gene arrangements [97]. The Tn554-like transposons introduces the arsenate resistance operon (arsCBADR) into the *L. monocytogenes* chromosome, but, as yet, it has not been characterized into a deeper in detail [98].

## 3. Discussion

Protein extracts from nine isolated *Listeria* species were analyzed and characterized by LC– ESI-MS/MS. The analysis identified 2990 non-redundant peptides, of which 395 represent proteins that act as either listerial virulence factors, toxins, or antibiotic resistance peptides, as well as tolerance proteins involved in bacterial resistance to toxic substances. Other proteins identified include colonization and immune evasion factors, polypeptides associated with antimicrobial production, ABC transporters, and other transporters associated with virulence factors. The *Listeria* strains used were previously characterized, in several studies, using proteomic techniques involving MALDI–TOF MS and LC– ESI-MS/MS [15,16,18,19,20]. In addition, the analyses here reported involved an in-depth study of the antimicrobial and virulence factors present in the strains, using shotgun proteomics tools. The rapid and accurate identification and potential pathogenicity characterization of pathogenic bacteria, including listerial species, is an essential issue to maintain a good quality food chain [99].

As mentioned above, Li2 and Li4 are the *Listeria* strains containing the highest number of virulence-related peptides identified (with 116 and 73 peptides, respectively), indicating that these represent the most pathogenic bacteria, as compared with the other strains analyzed. The methodology described here allows determination of specific virulence factors as displayed by the bacteria in situ, within a particular environment; hence it permits determination of the actual virulence in the bacterial strains contaminating the food chain. On the other hand, strain isolation and subsequent growth in laboratory conditions does not always reflect the characteristics displayed by a particular strain in a different environment.

All pathogen adaptations to either environmental stresses or sublethal concentrations of antimicrobial agents contribute to the development of antimicrobial resistances. In fact, understanding the full mechanism that permits *L. monocytogenes* to survive these adverse conditions, including pathogen virulence, antimicrobial resistance mechanisms and adaptation to environmental stress, is vital for the management and control of this pathogen, as it would aid in the development of novel, efficient, antimicrobial agents and in the development of biocontrol methods [100]. Unfortunately, the last decades have seen an increase in antibiotic resistance in listerial strains isolated from food products. Based on the clinical experience accumulated from other virulent bacterial strains, such as *Staphylococcus* and *Pseudomonas*, *L. monocytogenes* would likely become, in the near future, another multiresistant bacteria causing multitude of human deaths and increasing the rate of hospitalization. Although the number of *Listeria* strains exhibiting multidrug resistance is currently low, they are known to be progressively increase, by reasons yet unknown [7]. That is why continuous monitoring, and source tracking are currently imperative [101].

The studied *Listeria* strains contain many peptides involved in their resistome, including resistances to penicillin that are mediated by beta-lactamases and MarR protein, M56 peptidase family and penicillin-binding proteins. A variety of different drugs transports were also found, such as the multidrug ABC transporters, multidrug efflux MFS transporter, RND superfamily drug exporter, multidrug export protein mepA, and multidrug efflux SMR transporter. More peptides involved in other resistances such as gentamicin, kanamycin, tobramycin, neomycin, fosfomycin and apramycin were also found. As mentioned above, the prevalence of antibiotic resistances, including multiple antibiotic resistances, within the genus *Listeria* spp. is most concerning. Moreover, resistance to penicillins is increasing all over the world [102]; this makes it even more essential that pathogenic bacterial strains are quickly characterized and identified in foodstuffs as well as in clinics, in order to provide the appropriate antimicrobial treatment. This publication shows that bacterial characterization can be quickly achieved by the use of LC–ESI–MS/MS. As a matter of fact, there is an urgent need for novel therapies to both treatment and listeriosis prevention. As it is known, bacteriocins are active against antibiotic resistant bacterial strains, and accordingly they could be used, either independently or in combination with other antimicrobials. Moreover, a bacteriocin (belonging to the Lactococcin 972 family) has been found here. Deeper analysis should be performed however in the field of novel antimicrobials against pathogenic Listerial species.

In addition, some studies have demonstrated that an average of 54% of the *L. monocytogenes* strains may harbor plasmids. Schmitz-Esser and coworkers have described how plasmids were significantly more abundant in *L. monocytogenes* strains isolated from food and/or food production environments, compared to clinical strains, and in turn providing important advantages for survival in food or associated environments [103]. As the presence of mobile genetic elements is considered a major mechanism of antibiotic resistance acquisition for *L. monocytogenes* [7], the characterization of many resistances and tolerances that are maintained by plasmids in *Listeria* were determined from food products. A multidrug resistant (MDR) foodborne *L. monocytogenes* isolate was recovered by Li and colleges in 2016. It was observed how a plasmid carrying *cat*, *ermB*, and *tetS* genes, increased susceptibility to several antibiotics. This study demonstrated that the multi-drug resistant plasmid from *L. monocytogenes* is susceptible to be uptaken by *Streptococcus mutans* in a natural transformation fashion, and this resulted in the acquiring of multiple resistances as far as the transconjugants are concerned [104]. Besides, benzalkonium chloride (BC) and cadmium (Cd) tolerances are produced by bcrABC and cadAC genes-harboring plasmid in *L. monocytogenes,* isolated from food products of animal origin. The study also demostrated the potential horizontal transferability of this bcrABC cassette-harboring plasmid from a Gram-positive bacterium such as *L. monocytogenes* to the Gram-negative *E. coli*. Moreover, sub-lethal concentration of benzalkonium chloride (BC) may act as a selective pressure marker for *L. monocytogenes* strains that exhibited higher tolerance to some antibiotics as well as other toxic compounds [105]. In addition, it has been determined that a non-conjugative multiresistance plasmid (pNH1) from *L. monocytogenes* harbored nine antimicrobial resistance genes, including a multiresistance gene cluster. Sequence analysis revealed that the multiresistance gene cluster is part of a novel transposon, designated as Tn665. The association of the multiresistance gene cluster with an active transposon will support its persistence and dissemination among *L. monocytogenes* and potentially also other Gram-positive bacteria [106]. Other transposons determined for *Listeria* strains in this study such as Tn3 [30] and Tn554 [98] and Tn555 have been determined to be involved in antibiotic resistances and others.

Finally, many peptides corresponding to proteins that play a role in colonization and immune evasion of pathogenic *Listeria* strains have been identified in this study. These proteins play a crucial role in bacterial internalization into mammalian cells, during the course of infection, thus identification of microbial peptides may provide a way of characterization of the pathogen, even at the serotype level. MALDI-TOF MS has been used to identify the lineage of *L. monocytogenes* strains, but the method did not display enough potential to be useful in serotyping [98]. The GtcA protein (identified here in Li5 strain, serovar 1/2) has been reported to be serotype-specific for some of the *L. innocua* and *L. monocytogenes* strains [82,83,84]. Within *L. monocytogenes*, the *gltA-gltB* cassette is found only in strains belonging to serotypes 4b complex, 4d and 4e. Although *gtcA* was originally believed to be exclusive of serogroup 4, it was soon discovered that it was also present in serotype 1/2a, which harbors a similar, yet divergent, allelic form (sharing a homology of 80% at the nucleotide level and 82% in the amino acid sequence) and that is also located in an equivalent locus [100].

The precise proteomic method implemented in the present study represents a useful step for further analyses of pathogenic bacteria, as it offers considerable advantages over traditional approaches, in terms of speed and reliability, without the need for full genomic sequencing and analysis.

## 4. Materials and Methods

### 4.1. Bacterial Strains

Table 1 summarizes the nine *Listeria* strains used in this study, as well as their classification as either pathogenic or spoilage species. Eight reference bacterial strains were obtained from the Spanish Type Culture Collection, while the FBUNI strain was procured from the bacterial collection belonging to the Department of Microbiology, Faculty of Pharmacy, University of Santiago de Compostela (USC). The strains were previously analyzed by both MALDI-TOF MS and phylogenetic analyses on the 16S rRNA gene, and the genetic results compared to the proteomic data [107]. The *Listeria* strains were grown in Brain Heart Infusion (BHI, Oxoid Ltd., Hampshire, UK), at 31 °C for 24 h. Bacterial cultures were then transferred to plate count agar (PCA, Oxoid Ltd., Hampshire, UK) and subjected to further incubation at 31 °C for 24 h.

### 4.2. Protein Extraction

Protein extraction was done as indicated previously [23,108]. In short, a fresh inoculation loop of bacterial culture was resuspended in 100 μL of a solution with 50% acetonitrile (ACN; Merck, Darmstadt, Germany) and 1% aqueous trifluoroacetic acid (TFA; Acros Organics, NJ, USA). After vortexing and centrifuging, the supernatant was further treated with a lysis buffer consisting of 60 mM Tris-HCl pH 7.5, 1% lauryl maltoside, 5 mM phenylmethanesulfonyl fluoride (PMSF) and 1% dithiothreitol (DTT). The supernatant was transferred to a new tube and the amount of protein determined by the bicinchoninic acid method (Sigma Chemical Co. St. Louis, MO, USA). All experiments were performed in triplicate.

### 4.3. Peptide Sample Preparation

Protein extracts were solubilized and further digested with trypsin, as reported previously [109]. For doing so, 100 μg of protein was dried in a SpeedVac (CentriVap, Labconco Co., Kansas, MO, USA), resuspended in 25 μL of denaturation buffer, (8 M urea in 25 mM ammonium bicarbonate, pH 8.0), and sonicated for 5 min. Then, the addition of DTT followed, at a final concentration of 10 mM, and incubated at 37 °C for 1 h. Alkylation was easily achieved by addition of the appropriate amount of iodoacetamide (IAA), to a final concentration of 50 mM; the solution was dark-incubated for 1 additional hour at room temperature. The sample was diluted with 4 volumes of 25 mM ammonium bicarbonate (pH 8.0), in order to reduce the urea concentration. The final step included trypsin digestion (Promega, Madison, WI, USA), being the protease:protein ratio of 1:100. The incubation was performed overnight at 37 °C.

### 4.4. Shotgun LC–ESI–MS/MS Analysis

The peptide digests prepared as shown before were treated with formic acid (FA) (~pH 2), desalted in a C18 MicroSpin™ column (The Nest Group, Southborough, MA, USA) and finally analyzed by LC–ESI–MS/MS [104], using a Proxeon EASY-nLC II Nanoflow system (Thermo Fisher Scientific, San Jose, CA, USA) coupled to an LTQ-Orbitrap XL mass spectrometer (Thermo Fisher Scientific) [23,110]. Peptide separation (2 μg) was done in a reverse-phase (RP) column (EASY-Spray column, 50 cm × 75 μm ID, PepMap C18, 2 μm particles, 100 Å pore size, Thermo Fisher Scientific) equipped with a 10 mm precolumn (Accucore XL C18, Thermo Fisher Scientific). Elution from the column was accom by means of a linear gradient from 5 to 35% solvent B (solvent A: 98% water, 2% ACN, 0.1% FA; solvent B: 98% ACN, 2% water, 0.1% FA), for 120 min at a flow rate of 300 nL/min. Electrospray ionization was carried out with a spray voltage of 1.95 kV at a capillary temperature of 230 °C. Peptides were analyzed in positive mode (1 μscan; 400 to 1600 amu), followed by 10 data-dependent collision-induced dissociation (CID) MS/MS scans (1 μscan), using an isolation width of 3 amu and a normalized collision energy of 35%. After the second fragmentation event, dynamic exclusion was set for 30 s, and ions with unassigned charge state were excluded from MS/MS analysis.

### 4.5. LC–ESI–MS/MS Data Processing

The MS/MS spectra obtained by LC–ESI–MS/MS were analyzed using the program SEQUEST-HT (Proteome Discoverer 2.4, Thermo Fisher Scientific) and compared to the *Listeriaceae* UniProt/TrEMBL protein database (containing 267,496 protein sequence entries). MS/MS spectra were searched using fully tryptic cleavage constraints, and up to two missed cleavage sites were allowed. Tolerance windows were set at 10 ppm, for precursor ions, and 0.06 Da for MS/MS fragment ions. The variable modifications allowed were: (M*) methionine oxidation (+15.99 Da) and protein N-terminal acetylation (+42.0106 Da). Carbamidomethylation of Cys (+57.02 Da) (C*) was considered as fix modification. Percolator algorithm [111] was used to validate the results as well as for statistical analysis. The peptide false discovery rate (FDR was always kept at less than 1%.

## Figures and Tables

**Table 1:** *Listeria* strains used in this study. Total virulence factor peptides represent the number of peptides identified by LC–ESI-MS/MS. Pathogenicity data corresponds to the risk group of each analyzed strain. ATCC—American Type Culture Collection; CECT—Spanish Type Culture Collection; NCTC—The National Collection of Type Cultures of Public Health England.

Sample	Species	Strain	Culture Collection Number	Source	Pathogenicity	Variety	NCBI Accession Number	Total Virulence Factor Peptides
Li1	*Listeria welshimeri*	ATCC 35897	CECT 919	Decaying vegetation	Group 1	Serovar 6a	NZ_LT906444.1	17
Li2	*Listeria seeligeri*	ATCC 35967	CECT 917	Soil	Avirulent Group 1	Serovar 1/2b	DQ065845 NC_013891.1	116
Li3	*Listeria innocua*	ATCC 33090	CECT 910	Cow brain	Group 1	Serovar 6a	X98527	55
Li4	*Listeria monocytogenes*	LIS FBUNI	USC	Dairy product	-	-	-	73
Li5	*Listeria monocytogenes*	ATCC 15313	CECT 4031	Rabbit	Group 2	Serovar 1/2	AJ515512 NC_003210.1	15
Li6	*Listeria monocytogenes*	ATCC 19114	CECT 934	Brain of sheep with circling disease	Group 2	Serovar 4a	JF967620.1	18
Li7	*Listeria monocytogenes*	ATCC 13932	CECT 935	Spinal fluid of child with meningitis	Group 2	Serovar 4b	JF967617.1	33
Li8	*Listeria monocytogenes*	NCTC 11994	CECT 4032	Associated with case of meningitis after eating soft cheese	Group 2	Serovar 4b	AJ508749.1	37
Li9	*Listeria ivanovii*	ATCC 19119	CECT 913	Sheep	Group 2	Serovar 5	X98528 NZ_CP009577.1	31

**Table 2:** Proteins corresponding to bacterial resistance to antibiotics, antimicrobial related proteins and other virulence factors, identified in the *Listeria* strains analyzed.

Function	Protein
Antibiotic resistance	Beta-lactamase
	Metallo-beta-lactamase
	PbpX beta-lactamase-like superfamily
	Bleomycin resistance protein
	Aminoglycoside N(3)-acetyltransferase
	Glyoxalase/bleomycin resistance protein/dioxygenase
	MarR family transcriptional regulator
	Peptidase M56
	FosX/FosE/FosI family fosfomycin resistance thiol transferase
	GNAT family acetyltransferase
	PhzF family phenazine biosynthesis protein
	TetR
	VanZ family protein O
	YdeI (YjbR/CyaY-like superfamily)
	Tetronasin resistance transmembrane protein
	Penicillin-binding protein
Additional resistances and tolerances	AIPR protein
	Chaperone protein ClpB
	Chaperone protein DnaJ
	Tellurite resistance protein TelA
	Cass2 domain-containing protein
	Copper resistance protein
	MerR family transcriptional regulator
	Organic hydroperoxide resistance protein
	Two-component sensor histidine kinase response to antimicrobials
	Quaternary ammonium compound-resistance protein sugE
	Quinolone resistance protein norB
	Heat shock protein 70
	Hsp20/alpha crystallin family protein
	General stress protein
	OsmC family protein
	PadR family transcriptional regulator
	RsbU protein
	Universal stress protein
Antimicrobial compounds production	Lactococcin 972 family bacteriocin
	Antibiotic biosynthesis monooxygenase
Toxins	Toxin zeta
	LXG domain-containing protein
	HicA toxin
	Type II toxin-antitoxin system RelE/ParE family toxin
	Type II toxin-antitoxin system PemK/MazF family toxin
Host colonization and immune evasion	Autoinducer 2 transporter
	Viral enhancin protein
	Invasion associated secreted endopeptidase
	Peptidase_C1B
	Aminopeptidase ysdC
	Beta-Ala-Xaa dipeptidase
	Peptidase M23
	Peptidase M16
	Peptidase M23
	M23 family metallopeptidase
	Peptidoglycan DD-metalloendopeptidase family protein M22
	Peptidoglycan DD-metalloendopeptidase family protein M23
	Peptidase_M22
	M42 family peptidase
	Enhancing factor (Viral) Peptidase M60
	Peptidase M60
	Peptidase T
	Peptidase T
	Peptidase_M75
	Peptidase C60 sortase
	Membrane protein with peptidase activity M56
	Tripeptide aminopeptidase M20
	Peptidase SA1530
	Peptidase SA1531
	NLP/P60 family domain
	Invasion-associated endopeptidase p60
	Peptidoglycan endopeptidase P60
	Peptidase P60
	Competence protein ComEC/Rec2
	Protein ComGA
	Peptidoglycan O-acetyltransferase
	ClpX protease
	ClpP protease
	ClpY protease
	Internalin A
	Internalin
	Internalin
	Internalin-J
	Class 1 internalin InlJ
	Class 3 internalin InlC
	Internalin B
	Capsular polysaccharide synthesis enzyme
	Capsular biosynthesis protein
	Capsular exopolysaccharide family protein
	1-phosphatidylinositol- specific phospholipase C (PI-PLC)
	Chemotaxis protein CheA
	Cna B-type domain-containing protein
	N-acetylmuramoyl-L-alanine amidase
	D-alanyl-D-alanine carboxypeptidase
	Muramidase-2; Autolysin
	LysM domain-containing protein
	LysR family transcriptional regulator
	Autolysin LytC
	Lysis protein
	Superoxide dismutase
	Type VI secretion protein
	Type VII secretion protein EssA
	Type VII secretion protein EssB
	Putative type IV secretion system VirB4 component
	Mga protein
	Collagen-binding protein (adhesin)
	Adhesion lipoprotein
	Adhesin
	Intercellular adhesion protein R
	ESAT-6-like protein
	Insulinase
	Rgg family transcriptional regulator
	Protein DltD D-alanyl-lipoteichoic acid biosynthesis protein DltD
	Putative glycosyl/glycerophosphate transferases involved in teichoic acid biosynthesis
	Wall teichoic acid glycosylation protein GtcA
	Teichoic acid biosynthesis domain-containing protein
	Teichoic acid/polysaccharide export protein
ABC transporters	Fluoroquinolones export ATP-binding protein Rv2688c/MT2762
	Antibiotic ABC transporter ATP-binding protein
	Petrobactin ABC transporter substrate-binding protein YclQ
	Bacitracin ABC transporter BceA
	Antibiotic ABC_2_transport permease
	Multidrug ABC export ATP-binding/permease protein
	Multidrug ABC transporter permease/ATP-binding protein
	Multidrug resistance ABC transporter ATP-binding/permease protein YheI
	Multidrug ABC transporter ATP-binding protein
	Spermidine/putrescine ABC Transporter PotA
	Siderophore ABC transporter
	ABC transporter ATP-binding protein
	ABC transporter domain-containing protein
	ABC transporter permease
	ABC transporter substrate-binding protein
	ABC transporter permease
	ABC-2 family transporter protein
	Peptide ABC transporter
	Oligopeptide ABC transporter binding protein AppA
	ABC-type methionine transporter MetN
	Amino acid ABC transporter
	Di-tripeptide-proton ABC symporter
	Glutamine ABC transporter ATP-binding protein
	Glycine/betaine ABC transporter
	Amino acid ABC transporter ATP-binding protein
	Iron compound ABC transporter permease
	Macrolide ABC transporter
	Manganese ABC transporter
	Metal ABC transporter ATP-binding protein
	Zinc ABC transporter substrate-binding protein AdcA
	Putative high-affinity zinc ABC transporter (Zn(II)-binding lipoprotein)
	Pheromone ABC transporter
	Thiol reductant ABC exporter subunit CydC
Other transporters	Arsenic transporter
	Arsenical pump-driving ATPase
	Cadmium, zinc, and cobalt-transporting ATPase
	Chloramphenicol/florfenicol efflux MFS transporter
	EmrB multiple drug resistance efflux pump MFS transporter
	Multidrug efflux SMR transporter
	Multidrug export protein mepA
	Multidrug efflux MFS transporter
	RND superfamily drug exporter
	Putrescine importer PuuP
	Integrase
	Recombinase
	IS21 family transposase
	IS3 family transposase
	IS30 family transposase
	Transposase B of Tn554
	Transposase B of Tn555
	Tn3 family transposase
	IS200/IS605 family transposase
	IS30 family transposase
	Transposase IS116/IS110/IS902 family protein
	Mutator family transposase
	Plasmid recombination enzyme
	Plasmid pRiA4b ORF-3 family protein
	Plasmid pRiA4b ORF-3 family protein
	Plasmid mobilization relaxosome protein MobC
	Bacillus transposase protein
	Phage infection protein, YhgE

## Data Availability

Not applicable.

## Supplementary Materials

The following are available online at https://www.mdpi.com/article/10.3390/ijms22158141/s1, Excel data sheet in Supplementary Data S1: Complete list of non-redundant peptides identified for the complete dataset and for each *Listeria* strain. Tables S1–S6: Peptides corresponding to virulence factors, identified in the analyzed *Listeria* strains.
