# Supplementary material for: Proteomic Characterization of Antibiotic Resistance in Listeria and Production of Antimicrobial and Virulence Factors
Source: Int J Mol Sci. 2021 Jul 29;22(15):8141. doi: 10.3390/ijms22158141 (PMC8348566; doi:10.3390/ijms22158141)
Supplement: Supplementary file 1 [file ijms-22-08141-s001.zip › Suplemental data Table S1-S6.pdf]

International Journal of *Molecular Sciences*

MDPI

Article

Proteomic Characterization of Antibiotic Resistance in *Listeria* and Production of Antimicrobial and Virulence Factors

Ana G. Abril [1], Mónica Carrera [2,*], Karola Böhme [3], Jorge Barros-Velázquez [4], Pilar Calo-Mata [4], Angeles Sánchez-Pérez [5] and Tomás G. Villa [1,*]

1 Departamento de Microbiología y Parasitología, Facultad de Farmacia, Campus Sur 15782, Universidad de Santiago de Compostela, 15782, Santiago de Compostela, Spain; anagonzalezabril@hotmail.com
2 Marine Research Institute (IIM), Spanish National Research Council (CSIC), Eduardo Cabello 6, 36208 Vigo, Spain
3 Agroalimentary Technological Center of Lugo, Montirón 154, 27002 Lugo, Spain; KarolaBoehme@gmx.de
4 Departamento de Química Analítica, Nutrición y Bromatología, Área de Tecnología de los Alimentos, Facultad de Veterinaria, Campus Lugo. Universidad de Santiago de Compostela, 27002, Santiago de Compostela, Spain; jorge.barros@usc.es (J.B.-V.); p.calo.mata@usc.es (P.C.-M.)
5 Sydney School of Veterinary Science, Faculty of Science, University of Sydney, Sydney, NSW 2006, Australia; angelines2085@icloud.com
* Correspondence: mcarrera@iim.csic.es (M.C.); tomas.gonzalez@usc.es (T.G.V.)

Supplementary Materials

Table S1. Peptides corresponding to bacterial resistance to antibiotics and other toxic substances, identified in the *Listeria* strains analyzed.

Function	Protein	Sequence	Strain
Antibiotic resistance	Beta-lactamase	PQKGSLEQLQK	*Li7- Listeria monocytogenes*
	Beta-lactamase	VIDLQQK	*Li4- Listeria monocytogenes*
	Metallo beta lactamase Lin0319	NKRYYTVL	*Li8- Listeria monocytogenes*
	Metallo-beta-lactamase	LNEAITLR	*Li2- Listeria seeligeri*
	PbpX beta-lactamase-like superfamily	KPDNPVK	*Li7- Listeria monocytogenes*
	Bleomycin resistance protein	DAGLEIEEFVDR	*Li2- Listeria seeligeri*
	Aminoglycoside N(3)-acetyltransferase	GKAALLKDGK	*Li4- Listeria monocytogenes*
	Aminoglycoside N(3)-acetyltransferase	ANATSISTLKAK	*Li8- Listeria monocytogenes*
	Glyoxalase/bleomycin resistance protein/dioxygenase	RVTSKAEVAK	*Li2- Listeria seeligeri*
	Glyoxalase/bleomycin resistance protein/dioxygenase	NANLVMDGVK	*Li4- Listeria monocytogenes*
	MarR family transcriptional regulator	DSIHRAKNPQ	*Li3- Listeria innocua*
	MarR family transcrip-	KMINNVEK	*Li2- Listeria*

tional regulator		*seeligeri*
MarR family transcriptional regulator	NISWLQYDIL	*Li1- Listeria welshmeri*
Peptidase M56	QEM*ADKMKK	*Li2- Listeria seeligeri*
FosX/FosE/FosI family fosfomycin resistance thiol transferase	IAFQIQSK	*Li7- Listeria monocytogenes*
GNAT family acetyltransferase	MIVGVATLEEYR	*Li7- Listeria monocytogenes*
GNAT family acetyltransferase	RNSASIALLEK	*Li3- Listeria innocua*
GNAT family acetyltransferase	ISGAIIMNDLNT	*Li8- Listeria monocytogenes*
GNAT family acetyltransferase	IQDELKSLGY	*Li2- Listeria seeligeri*
PhzF family phenazine biosynthesis protein	INEEAATGTSNGALAC*YLYKNQYLQK	*Li2- Listeria seeligeri*
PhzF family phenazine biosynthesis protein	INEEAATGTSNGALAC*YLYQNQYLQK	*Li2- Listeria seeligeri*
TetR	M*AIFGTPDEKGAKK	*Li8- Listeria monocytogenes*
TetR	GQAEQSEMVSLVLNSVIL	*Li2- Listeria seeligeri*
TetR	VKQADVNR	*Li2- Listeria seeligeri*
TetR	RAVFYPPK	*Li5- Listeria monocytogenes*
TetR	EM*HPIMQAEK	*Li2- Listeria seeligeri*
TetR	TKPYSAI	*Li1- Listeria welshmeri*
TetR	RSM*DEMGEKAK	*Li4- Listeria monocytogenes*
TetR	VDFATKVVR	*Li2- Listeria seeligeri*
TetR	ELAIQQGVDTKY	*Li8- Listeria monocytogenes*
TetR	DSLINAHIEWIDQEAANGC*MFLRAK	*Li2- Listeria seeligeri*
VanZ family protein O	YAC*GVIK	*Li2- Listeria seeligeri*
YdeI (YjbR/CyaY-like superfamily)	M*TAGEM*TPK	*Li5- Listeria monocytogenes*
Tetronasin resistance transmembrane protein	WLPKLGK	*Li2- Listeria seeligeri*
Penicillin binding protein 2B	QPQLSGTETGGK	*Li2- Listeria seeligeri*
Penicillin binding protein 2B	VPKVEDNK	*Li2- Listeria seeligeri*

	Penicillin-binding protein 1A/1B	INGDQM*SPLL	*Li7- Listeria monocytogenes*
	Penicillin-binding protein 1A	LDQIGIDK	*Li6- Listeria monocytogenes*
	Penicillin-binding protein 1A/1B	QDKDKDK	*Li4- Listeria monocytogenes*
	Penicillin-binding protein 2	LMGINYQSGI	*Li4- Listeria monocytogenes*
	Penicillin-binding protein	NEIM*KEKLPG	*Li6- Listeria monocytogenes*
	Penicillin-binding protein	KEQYNSAVK	*Li4- Listeria monocytogenes*
	Penicillin-binding protein	EEQNLDMQVDALTKFGVEKIYK	*Li8- Listeria monocytogenes*
	Penicillin-binding protein	AEAFYDGPKK	*Li4- Listeria monocytogenes*
	Penicillin-binding protein	ENGGEIK	*Li6- Listeria monocytogenes*
	Penicillin-binding protein	ADGLVDEATRK	*Li6- Listeria monocytogenes*
	Penicillin-binding protein	AGVNLVTNGGISGGG	*Li3- Listeria innocua*
	Penicillin-binding protein	VSDPGGTAHALALNGKKLAAK	*Li3- Listeria innocua*
	Penicillin-binding protein	WVQPDSFVPLV	*Li2- Listeria seeligeri*
Additional resistances and tolerances	AIPR protein	ENLSETTK	*Li3- Listeria innocua*
	Chaperone protein ClpB	IPIVSGTNIEYGSS	*Li3- Listeria innocua*
	Chaperone protein ClpB	EIVSGKIMLHSS	*Li4- Listeria monocytogenes*
	Chaperone protein ClpB	IMLHSSVEID	*Li6- Listeria monocytogenes*
	Chaperone protein ClpB	TAIVEGLAQR	*Li4- Listeria monocytogenes*
	Chaperone protein ClpB	TKNNPVLIG	*Li4- Listeria monocytogenes*
	Chaperone protein ClpB	YRGEFEER	*Li4- Listeria monocytogenes*
	Chaperone protein ClpB	EAVGISKK	*Li4- Listeria monocytogenes*
	Chaperone protein DnaJ	RQDPTAPR	*Li4- Listeria monocytogenes*
	Chaperone protein DnaJ	RSFADTTGDK	*Li3- Listeria innocua*
	Tellurite resistance protein TelA	LVDM*LPENNRRQ	*Li4- Listeria monocytogenes*
	Tellurite resistance protein TelA	LQEADPDELAAR	*Li6- Listeria monocytogenes*

	Tellurite resistance protein TelA	QAEISQLK	*Li1- Listeria welshmeri*
	Cass2 domain-containing protein	DGVQTYLVAK	*Li4- Listeria monocytogenes*
	Copper resistance protein	QESNRIVSVK	*Li9- Listeria ivanovi*
	MerR family transcriptional regulator	KNAIELLSEK	*Li5- Listeria monocytogenes*
	MerR family transcriptional regulator	DTVVEKANEK	*Li4- Listeria monocytogenes*
	MerR family transcriptional regulator	NQILENSR	*Li9- Listeria ivanovi*
	MerR family transcriptional regulator	AMLNTLDQTIK	*Li4- Listeria monocytogenes*
	Organic hydroperoxide resistance protein	LYETTVINTGGR	*Li6- Listeria monocytogenes*
	Two-component sensor histidine kinase response to antimicrobials	KEQYFMPENEK	*Li5- Listeria monocytogenes*
	Quaternary ammonium compound-resistance protein sugE	VIGAVGLK	*Li4- Listeria monocytogenes*
	Quinolone resistance protein norB	QILAIGELK	*Li2- Listeria seeligeri*
	Heat shock protein 70	IAGLEVER	*Li4- Listeria monocytogenes*
	Hsp20/alpha crystallin family protein	ADLPGIKK	*Li2- Listeria seeligeri*
	General stress protein	IDILVNVAGEQH	*Li9- Listeria ivanovi*
	General stress protein	KASEVSGAAK	*Li4- Listeria monocytogenes*
	General stress protein	SSQLKDSANDFVGVVK	*Li2- Listeria seeligeri*
	OsmC family protein	EMGFSPVQ	*Li7- Listeria monocytogenes*
	PadR family transcriptional regulator	KLEKAGFLVFHK	*Li6- Listeria monocytogenes*
	RsbU protein	GALAPSEM*LRNL	*Li7- Listeria monocytogenes*
	Universal stress protein	DGSNESEAALR	*Li9- Listeria ivanovi*
	Universal stress protein	NEFADIARRAGVAK	*Li3- Listeria innocua*

M* methionine oxidation; C* carbamidomethylation of Cys.

Table S2. Peptides corresponding to antibacterial compounds and proteins involved in antibacterial production, identified in the *Listeria* strains analyzed.

Function	Protein	Sequence	Strain
Antimicrobial compounds production	Lactococcin 972 family bacteriocin	DSLMKANETGEK	*Li2- Listeria seeligeri*
	Antibiotic biosynthesis monooxygenase	KVTGHVVGGHQR	*Li8- Listeria monocytogenes*

Table S3. Peptides corresponding to bacterial toxicity, identified in the *Listeria* strains analyzed.

Function	Protein	Sequence	Strain
Toxins	Toxin zeta	YVMAVPK	*Li9- Listeria ivanovi*
	LXG domain-containing protein	EQINLLEEIK	*Li7- Listeria monocytogenes*
	LXG domain-containing protein	SITGKTDDGR	*Li3- Listeria innocua*
	LXG domain-containing protein	PQEIIDYFMK	*Li4- Listeria monocytogenes*
	LXG domain-containing protein	SVLENLKK	*Li4- Listeria monocytogenes*
	LXG domain-containing protein	M*EKIIEKLYGK	*Li3- Listeria innocua*
	LXG domain-containing protein	WNVELSKVEKLQ	*Li4- Listeria monocytogenes*
	LXG domain-containing protein	KSQVDDFVEHGKVLSQNEGEK	*Li2- Listeria seeligeri*
	HicA toxin	PITPKKM*AR	*Li6- Listeria monocytogenes*
	Type II toxin-antitoxin system RelE/ParE family toxin	NGM*LVAQR	*Li2- Listeria seeligeri*
	Type II toxin-antitoxin system PemK/MazF family toxin	GSLVM*VDFGVNVGNEISG	*Li2- Listeria seeligeri*
	Toxin-antitoxin system antitoxin	QM*KDESISFDEMKK	*Li2- Listeria seeligeri*

M* methionine oxidation; C* carbamidomethylation of Cys.

Table S4. Peptides corresponding to proteins involved in host colonization and immune evasion, identified in the *Listeria* strains analyzed.

Function	Protein	Sequence	Strain
Host colonization and immune evasion	Autoinducer 2 transporter	DKKQTTDK	*Li9- Listeria ivanovi*
	Autoinducer 2 transporter	TVQEFM*AK	*Li2- Listeria seeligeri*
	Viral enhancin protein	IRVHHEEGEARLSVQNR	*Li3- Listeria innocua*
	Invasion associated secreted endopeptidase	EEVKQETAPQ	*Li1- Listeria welshmeri*
	Invasion associated secreted endopeptidase	QTTQTNTAETTSVK	*Li2- Listeria seeligeri*
	Peptidase	QGSSRDVTGK	*Li2- Listeria seeligeri*
	Peptidase_C1B	KDAVALR	*Li4- Listeria monocytogenes*
	Aminopeptidase ysdC	SAIAAKVGDAEGPK	*Li2- Listeria*

			seeligeri
	Beta-Ala-Xaa dipeptidase	DDLLEDLQN	*Li2- Listeria seeligeri*
	Peptidase M23	KLNEGLK	*Li2- Listeria seeligeri*
	Peptidase M16	LGKISELEIE	*Li4- Listeria monocytogenes*
	Peptidase M23	EELQSQQEQLQ	*Li4- Listeria monocytogenes*
	M23 family metallopeptidase	LGGLTTNC*SQ	*Li3- Listeria innocua*
	Peptidoglycan DD-metalloendopeptidase family protein M22	NAEELKKLEK	*Li9- Listeria ivanovi*
	Peptidoglycan DD-metalloendopeptidase family protein M23	LNVSQGQSVKAGDV	*Li4- Listeria monocytogenes*
	Peptidase_M22	WLEARKK	*Li4- Listeria monocytogenes*
	M42 family peptidase	VGAEDGPKIM*IA	*Li3- Listeria innocua*
	Peptidase	QLWDQAPDAAI	*Li4- Listeria monocytogenes*
	Peptidase	QTIENEEPPEEK	*Li5- Listeria monocytogenes*
	Enhancing factor (Viral) Peptidase M60	LKLSVGNADQ	*Li1- Listeria welshmeri*
	Peptidase M60	GEGPKDLTEL	*Li8- Listeria monocytogenes*
	Peptidase M60	KNM*LHLDIK	*Li9- Listeria ivanovi*
	Peptidase T	QDIVLNK	*Li2- Listeria seeligeri*
	Peptidase T	KAIVLNK	*Li2- Listeria seeligeri*
	Peptidase_M75	DELATVEKDIAAK	*Li2- Listeria seeligeri*
	Peptidase C60 sortase	KGVTGERR	*Li8- Listeria monocytogenes*
	Membrane protein with peptidase activity M56	SIGQSQTKFVIR	*Li2- Listeria seeligeri*
	Tripeptide aminopeptidase M20	IDSETTANIGI	*Li4- Listeria monocyto-*

			genes
	Peptidase SA1530	KAIAKVR	*Li2- Listeria seeligeri*
	Peptidase SA1531	ISQAHSGM*TGK	*Li2- Listeria seeligeri*
	NLP/P60 family domain	KTM*SAPK	*Li8- Listeria monocytogenes*
	Invasion-associated endopeptidase p60	LLDELSRNR	*Li8- Listeria monocytogenes*
	Invasion-associated endopeptidase p60	SGDTLSKIAS	*Li2- Listeria seeligeri*
	Peptidoglycan endopeptidase P60	RDATEQSFTGK	*Li2- Listeria seeligeri*
	Peptidase P60	SLQADFDKTGK	*Li5- Listeria monocytogenes*
	Peptidase P60	ATALREATDANVGQQTTNT	*Li7- Listeria monocytogenes*
	Peptidase P60	ATALREATDANVGQQTTNT-NASSNSK	*Li7- Listeria monocytogenes*
	Peptidase P60	AMQKTSNSNAYLE	*Li1- Listeria welshmeri*
	Peptidase P60	KLNEDIKSINER	*Li5- Listeria monocytogenes*
	Peptidase P60	SLQGEFDKTGK	*Li3- Listeria innocua*
	Peptidoglycan endopeptidase P60	MYFVDNNNEKD	*Li8- Listeria monocytogenes*
	Competence protein ComEC/Rec2	ELLM*EK	*Li6- Listeria monocytogenes*
	Protein ComGA	MPKQILANLLQ	*Li1- Listeria welshmeri*
	Peptidoglycan O-acetyltransferase	QVVNDTLK	*Li9- Listeria ivanovi*
	Clp protease	DGQGNVINFK	*Li4- Listeria monocytogenes*
	Clp protease ClpX	VIEVLSR	*Li4- Listeria monocytogenes*
	Clp protease ClpX	KAIDLM*DEIGSK	*Li9- Listeria ivanovi*
	ClpP protease	TGQPYEVIAR	*Li3- Listeria*

			innocua
	ClpP protease	AYDIYSR	*Li1- Listeria welshmeri*
	Clp protease	MSPVAMMMIHNPVTQTWG-DRVDMQK	*Li2- Listeria seeligeri*
	Clp protease	EPAVAKPESK	*Li6- Listeria monocyto-genes*
	ClpY protease	SQNGDGNAQVSR	*Li2- Listeria seeligeri*
	Clp protease	IASKEGGGNAQVSQEGVQR	*Li2- Listeria seeligeri*
	Internalin A	EAGNLLTEPAK	*Li2- Listeria seeligeri*
	Internalin A	DVGSLDDLPAL	*Li3- Listeria innocua*
	Internalin A	YLGTFAGSALK	*Li1- Listeria welshmeri*
	Internalin	WNLQAYTK	*Li3- Listeria innocua*
	Internalin	SDVTDIITQDDINAATSLG	*Li9- Listeria ivanovi*
	Internalin-J	SQVITTYQTKD	*Li2- Listeria seeligeri*
	Internalin-J	TLLTETANK	*Li2- Listeria seeligeri*
	Internalin-J	ISDDLIR	*Li4- Listeria monocyto-genes*
	Class 1 internalin InlJ	ESKLPSTGDIV	*Li8- Listeria monocyto-genes*
	Class 1 internalin InlJ	IEASIEQIEK	*Li8- Listeria monocyto-genes*
	Class 3 internalin InlC	FDGTVTQPILN	*Li7- Listeria monocyto-genes*
	Internalin A	EITYTVNTLK	*Li2- Listeria seeligeri*
	Internalin B	TLSVYAGK	*Li4- Listeria monocyto-genes*
	Internalin	KISGATARK	*Li2- Listeria seeligeri*
	Capsular polysaccharide synthesis enzyme	KNDKLEVETIR	*Li4- Listeria monocyto-genes*
	Capsular polysaccharide synthesis enzyme	ITTAKIIFDMH	*Li9- Listeria ivanovi*

	Capsular biosynthesis protein	MKGSNIM*AEEAL	*Li2- Listeria seeligeri*
	Capsular exopolysaccharide family protein	FTSANKGEGKTFcVS	*Li4- Listeria monocytogenes*
	1-phosphatidylinositol-specific phospholipase C (PI-PLC)	KAHSIKNSYR	*Li7- Listeria monocytogenes*
	Chemotaxis protein CheA	TFGLIIKD	*Li4- Listeria monocytogenes*
	Cna B-type domain-containing protein	MGVTGSVSKDAR	*Li4- Listeria monocytogenes*
	Cna B-type domain-containing protein	GENAILEK	*Li4- Listeria monocytogenes*
	Cna B-type domain-containing protein	KVVDVSK	*Li2- Listeria seeligeri*
	Cna B-type domain-containing protein	NQDGIRPVK	*Li3- Listeria innocua*
	Cna B-type domain-containing protein	KSGSVTPSGK	*Li4- Listeria monocytogenes*
	Cna B-type domain-containing protein	LQNGQTYGDPVK	*Li3- Listeria innocua*
	N-acetylmuramoyl-L-alanine amidase	LAFDVTSQVR	*Li5- Listeria monocytogenes*
	N-acetylmuramoyl-L-alanine amidase	LAVFAGHGGVDSGAV	*Li3- Listeria innocua*
	N-acetylmuramoyl-L-alanine amidase	RNSDKYVSLK	*Li7- Listeria monocytogenes*
	D-alanyl-D-alanine carboxypeptidase	AGSQLEAMFAAAK	*Li3- Listeria innocua*
	D-alanyl-D-alanine carboxypeptidase	MNTIISVAFAIALSVVQTV	*Li6- Listeria monocytogenes*
	D-alanyl-D-alanine carboxypeptidase	NADNALLVSVK	*Li4- Listeria monocytogenes*
	Muramidase-2; Autolysin	NENTPAK	*Li7- Listeria monocytogenes*
	LysM domain-containing protein	VQAGEGPEQVAAR	*Li3- Listeria innocua*
	LysM domain-containing protein	KAAASSHTVQ	*Li2- Listeria seeligeri*
	LysM domain-containing	AGGSHTVQAGDTLYS	*Li7- Listeria*

	protein		*monocytogenes*
	LysM domain-containing protein	GQAGAAAGVEKIK	*Li2- Listeria seeligeri*
	LysM domain-containing protein	QAGAAAGVEKIK	*Li2- Listeria seeligeri*
	LysM domain-containing protein	EEKAAPSTQKSTSSNEASTNSSSSA	*Li2- Listeria seeligeri*
	LysR family transcriptional regulator	IMVGAAETRGFE	*Li9- Listeria ivanovi*
	LysR family transcriptional regulator	AGNTTGKSVQK	*Li2- Listeria seeligeri*
	LysR family transcriptional regulator	QMILEGMGYTVISK	*Li4- Listeria monocytogenes*
	LytR family transcriptional regulator	FRKDAEGDFGR	*Li7- Listeria monocytogenes*
	LytR family transcriptional regulator	LLM*GSDARAGEK	*Li4- Listeria monocytogenes*
	LytR family transcriptional regulator	MEADGVIIAAA	*Li8- Listeria monocytogenes*
	LytR family transcriptional regulator	QDTPM*KLADGL	*Li7- Listeria monocytogenes*
	LytR family transcriptional regulator	EASLNDLEQLR	*Li6- Listeria monocytogenes*
	LytTR family transcriptional regulator	VYC*HPDNLAEVKNM*VMK	*Li2- Listeria seeligeri*
	LytTR family transcriptional regulator	VYC*HPDNLAEVKNMVM*K	*Li2- Listeria seeligeri*
	Autolysin LytC	EIGGQTYMVK	*Li1- Listeria welshmeri*
	Autolysin LytC	SDVDTLVTGK	*Li2- Listeria seeligeri*
	Lysis protein	ADAKAENDALRDDVAAGR	*Li2- Listeria seeligeri*
	Superoxide dismutase	HHNTYVTKL	*Li6- Listeria monocytogenes*
	Superoxide dismutase	HHNTYVTKLNEA	*Li3- Listeria innocua*
	Type VI secretion protein	VM*AERII	*Li2- Listeria seeligeri*
	Type VI secretion protein	EIKHNGFR	*Li2- Listeria seeligeri*
	Type VII secretion protein	GKM*EIKTDR	*Li2- Listeria*

	EssA		*seeligeri*
	Type VII secretion protein EssB	NITFPKSK	*Li7- Listeria monocytogenes*
	Putative type IV secretion system VirB4 component	DVIVTMDVGTQNSEKILE	*Li5- Listeria monocytogenes*
	Mga protein	EQKIQLGKT	*Li6- Listeria monocytogenes*
	Mga protein	QTFMNSEGPLLK	*Li3- Listeria innocua*
	Mga protein	MRLDLDILDTNVE	*Li2- Listeria seeligeri*
	Mga protein	VQNQNYVVQPTNK	*Li4- Listeria monocytogenes*
	Collagen-binding protein (adhesin)	IEDPKAEAK	*Li3- Listeria innocua*
	Collagen-binding protein (adhesin)	EGDGTDEPK	*Li3- Listeria innocua*
	Adhesion lipoprotein	PHEYEPLPKDIR	*Li3- Listeria innocua*
	Adhesin	PVLIPNK	*Li3- Listeria innocua*
	Intercellular adhesion protein R	NEIHM*NQAEK	*Li2- Listeria seeligeri*
	ESAT-6-like protein	MAGQIRM*SPAELRDR	*Li2- Listeria seeligeri*
	Insulinase	ELAYNNVLR	*Li4- Listeria monocytogenes*
	Rgg family transcriptional regulator	TSDSFNSAFYR	*Li2- Listeria seeligeri*
	Rgg family transcriptional regulator	GMLHMDKSIGEALR	*Li4- Listeria monocytogenes*
	Rgg family transcriptional regulator	TGVASGKCGFTTVSI	*Li1- Listeria welshmeri*
	Protein DltD D-alanyl-lipoteichoic acid biosynthesis protein DltD	IEGKLKDSR	*Li9- Listeria ivanovi*
	Putative glycosyl/glycerophosphate transferases involved in teichoic acid biosynthesis	PVIGTDVPGIKSVLAGSNGYLAK	*Li1- Listeria welshmeri*
	Wall teichoic acid glycosylation protein GtcA	ELIANFRKEH	*Li5- Listeria monocytogenes*
	Teichoic acid biosynthesis	FGEDVVLLIRM*H	*Li7- Listeria*

	domain-containing protein		*monocytogenes*
	Teichoic acid/polysaccharide export protein	GEMVTIPVK	*Li8- Listeria monocytogenes*

(M* methionine oxidation; C* carbamidomethylation of Cys).

Table S5. Peptides corresponding to transporters associated to virulence factors, identified in the *Listeria* strains analyzed.

Function	Protein	Sequence	Strain
ABC transporters	Fluoroquinolones export ATP-binding protein Rv2688c/MT2762	QDLEVLTSADAK	*Li8- Listeria monocytogenes*
	ABC transporter ATP-binding protein (Antibiotic resistance)	SGKELREDLNT	*Li8- Listeria monocytogenes*
	Antibiotic ABC transporter ATP-binding protein	SEALDIKKK	*Li4- Listeria monocytogenes*
	Bacitracin ABC transporter	ERDLQELEK	*Li8- Listeria monocytogenes*
	Petrobactin ABC transporter substrate-binding protein YclQ	GGLESVNEMIDEVGAVIK	*Li2- Listeria seeligeri*
	Bacitracin ABC transporter BceA	EAKLAVISK	*Li4- Listeria monocytogenes*
	Antibiotic ABC_2_transport permease	MLASQLEK	*Li6- Listeria monocytogenes*
	Multidrug ABC export ATP-binding/permease protein	KSGFKEV	*Li8- Listeria monocytogenes*
	Multidrug ABC export ATP-binding/permease protein	SRIQEALDMEPAIR	*Li4- Listeria monocytogenes*
	Multidrug ABC transporter ATP-binding protein	LLSLMSPLMM*L	*Li3- Listeria innocua*
	Multidrug ABC transporter ATP-binding protein	WDMNTEAKTILER	*Li2- Listeria seeligeri*
	Multidrug ABC transporter ATP-binding protein	QLQNSGMDLEDIG	*Li2- Listeria seeligeri*
	Multidrug ABC transporter permease/ATP-binding protein	M*AHATNDLK	*Li9- Listeria ivanovi*
	Multidrug resistance ABC transporter ATP-binding/permease protein YheI	EVVQKNI	*Li3- Listeria innocua*
	Multidrug ABC transporter ATP-binding protein	IETM*PESYDTIVGER	*Li2- Listeria seeligeri*
	Multidrug ABC transporter ATP-binding protein	SVGANGEIIR	*Li9- Listeria ivanovi*
	Spermidine/putrescine ABC Transporter PotA	LNDKVINEIPANK	*Li7- Listeria monocytogenes*
	Siderophore ABC transporter	AGNKYMDLGGLK	*Li5- Listeria monocytogenes*
	ABC transporter ATP-binding protein	LLNMPE	*Li2- Listeria seeligeri*
	ABC transporter ATP-binding protein	IKADLMLVSC*TQ	*Li8- Listeria monocytogenes*

	ABC transporter ATP-binding protein	GSGAIFDTGAIGAR	*Li1- Listeria welshmeri*
	ABC transporter ATP-binding protein	ELKATNIQ	*Li8- Listeria monocytogenes*
	ABC transporter ATP-binding protein	QLLLELQK	*Li2- Listeria seeligeri*
	ABC transporter ATP-binding protein	TDAADILGLTEYLER	*Li2- Listeria seeligeri*
	ABC transporter ATP-binding protein	TMLNPNGSVK	*Li8- Listeria monocytogenes*
	ABC transporter ATP-binding protein	TDLTAKVGALSGGQR	*Li2- Listeria seeligeri*
	ABC transporter ATP-binding protein	VGALSGGQR	*Li7- Listeria monocytogenes*
	ABC transporter ATP-binding protein	VLTVTGMAWTSVS	*Li3- Listeria innocua*
	ABC transporter ATP-binding protein	VGLTDSSKK	*Li7- Listeria monocytogenes*
	ABC transporter ATP-binding protein	AIAAASRVFR	*Li3- Listeria innocua*
	ABC transporter ATP-binding protein	GC*GVFRGVAR	*Li9- Listeria ivanovi*
	ABC transporter ATP-binding protein	GVSQFYGASR	*Li8- Listeria monocytogenes*
	ABC transporter ATP-binding protein	RQAANPKR	*Li7- Listeria monocytogenes*
	ABC transporter ATP-binding protein	MMVGRSVSFKTPK	*Li3- Listeria innocua*
	ABC transporter ATP-binding protein	KDM*PNK	*Li3- Listeria innocua*
	ABC transporter ATP-binding protein	RIGLTR	*Li4- Listeria monocytogenes*
	ABC transporter ATP-binding protein	LSTIENADLILV	*Li8- Listeria monocytogenes*
	ABC transporter ATP-binding protein	KKGEQVEVTASV	*Li4- Listeria monocytogenes*
	ABC transporter ATP-binding protein	M*TRTKADDVAVE	*Li2- Listeria seeligeri*
	ABC transporter ATP-binding protein	KNDATAAR	*Li2- Listeria seeligeri*
	ABC transporter ATP-binding protein	VEC*LRFM*GKLSNM*EK	*Li9- Listeria ivanovi*
	ABC transporter domain-containing protein	SFVARVDSR	*Li2- Listeria seeligeri*
	ABC transporter	DEVEKIKQAGK	*Li1- Listeria welshmeri*
	ABC transporter permease	TLIEDAVNDAAK	*Li3- Listeria innocua*
	ABC transporter permease	TTLIEDAVNDAA	*Li2- Listeria seeligeri*

	ABC transporter permease	TPTKIESKK	*Li2- Listeria seeligeri*
	ABC transporter permease	MTTVTKPSAAK	*Li2- Listeria seeligeri*
	ABC transporter permease	NKVDPLDAIR	*Li4- Listeria monocytogenes*
	ABC transporter permease	KDEKESVQK	*Li2- Listeria seeligeri*
	ABC transporter permease	ISNGEAELRSAKK	*Li9- Listeria ivanovi*
	ABC transporter permease	KAELKANEAK	*Li2- Listeria seeligeri*
	ABC transporter permease	EQGEQTLVAK	*Li2- Listeria seeligeri*
	ABC transporter permease	THDPVPLTNNGAVLS	*Li3- Listeria innocua*
	ABC transporter permease	GIKDGENVK	*Li2- Listeria seeligeri*
	ABC transporter permease	QGIFEK	*Li8- Listeria monocytogenes*
	ABC transporter permease	AVSPEFAEENK	*Li2- Listeria seeligeri*
	ABC transporter substrate-binding protein	SASKEDAGDGVTL	*Li2- Listeria seeligeri*
	ABC transporter substrate-binding protein	NKVEKASAVN	*Li2- Listeria seeligeri*
	ABC transporter substrate-binding protein	TFNENQPALK	*Li7- Listeria monocytogenes*
	ABC transporter substrate-binding protein	ENVSRIAH	*Li8- Listeria monocytogenes*
	ABC transporter substrate-binding protein	SDDNLTYTVK	*Li7- Listeria monocytogenes*
	ABC transporter substrate-binding protein	GFYFDAKKVR	*Li7- Listeria monocytogenes*
	ABC transporter substrate-binding protein	LISSGTYSPNEI	*Li8- Listeria monocytogenes*
	ABC transporter substrate-binding protein	DAKSTIEKR	*Li3- Listeria innocua*
	ABC transporter substrate-binding protein	YLEQAAGETPK	*Li3- Listeria innocua*
	ABC transporter substrate-binding protein	TLFSTM*TGIYLPDAGDVFLNGK	*Li3- Listeria innocua*
	ABC transporter permease	EAKLMQAK	*Li3- Listeria innocua*
	ABC-2 family transporter protein	IQTEESAK	*Li9- Listeria ivanovi*
	Peptide ABC transporter	AQEYWEKAK	*Li4- Listeria monocytogenes*
	Peptide ABC transporter	KLANPKNQANYF	*Li4- Listeria monocytogenes*

	Peptide ABC transporter	EALGIDSLSFE	*Li6- Listeria monocytogenes*
	Peptide ABC transporter	ANGSSALNGNVPAK	*Li3- Listeria innocua*
	Peptide ABC transporter	GKPVLALAASEPEEK	*Li3- Listeria innocua*
	Peptide ABC transporter	LADNSVSFR	*Li3- Listeria innocua*
	Peptide ABC transporter	AGKDTVFANK	*Li9- Listeria ivanovi*
	Peptide ABC transporter	AGKDTVFANKNIR	*Li5- Listeria monocytogenes*
	Peptide ABC transporter	AIALAIDKQ	*Li9- Listeria ivanovi*
	Peptide ABC transporter	DTVFANKNIR	*Li3- Listeria innocua*
	Peptide ABC transporter	ELVPDEGSLL	*Li8- Listeria monocytogenes*
	Peptide ABC transporter	FANKNIR	*Li3- Listeria innocua*
	Peptide ABC transporter	KAIALAIDKQ	*Li9- Listeria ivanovi*
	Peptide ABC transporter	KKPEELGI	*Li5- Listeria monocytogenes*
	Peptide ABC transporter	NDSSTFYIK	*Li9- Listeria ivanovi*
	Peptide ABC transporter	TVLSADYAAQNK	*Li7- Listeria monocytogenes*
	Peptide ABC transporter	DGKATPLANNSLR	*Li7- Listeria monocytogenes*
	Peptide ABC transporter	HAFGSPYSYK	*Li5- Listeria monocytogenes*
	Peptide ABC transporter	HILSLPMNFFA	*Li3- Listeria innocua*
	Oligopeptide ABC transporter -binding protein	NSLC*KYSNEK	*Li8- Listeria monocytogenes*
	Oligopeptide ABC transporter binding protein AppA	SPQQAQTTK	*Li2- Listeria seeligeri*
	ABC-type methionine transporter MetN	FGQVVEVQLPKEK	*Li8- Listeria monocytogenes*
	Amino acid ABC transporter	DDQAMADKINDGLK	*Li2- Listeria seeligeri*
	Amino acid ABC transporter	VMIPSFVNQFVMT	*Li2- Listeria seeligeri*
	Di-tripeptide-proton ABC symporter	GVTTQLAPR	*Li3- Listeria innocua*
	Glutamine ABC transporter ATP-binding protein	SAINIRK	*Li4- Listeria monocytogenes*
	Glutamine ABC transporter ATP-binding protein	IEFEGTNLTDK	*Li3- Listeria innocua*

	Glycine/betaine ABC transporter	NDAGGGFVAGI	*Li5- Listeria monocytogenes*
	Glycine/betaine ABC transporter	ANQAYVSVAR	*Li9- Listeria ivanovi*
	Glycine/betaine ABC transporter	WNKNNPDKVAK	*Li2- Listeria seeligeri*
	Glycine/betaine ABC transporter	C*SLPGLGGGAEGTIR	*Li7- Listeria monocytogenes*
	Amino acid ABC transporter ATP-binding protein	GSSGSGKSTLLR	*Li9- Listeria ivanovi*
	Iron compound ABC transporter permease	MVLLMASADR	*Li2- Listeria seeligeri*
	Iron compound ABC transporter permease	NDLADPGII	*Li2- Listeria seeligeri*
	Iron compound ABC transporter permease	DAALKAGYYAVSPE	*Li7- Listeria monocytogenes*
	Macrolide ABC transporter	PAQLSGGQQQR	*Li2- Listeria seeligeri*
	Manganese ABC transporter	SVSRDTDIPI	*Li3- Listeria innocua*
	Manganese ABC transporter	VPALFVETSVDSRSM*ESVSNETS	*Li2- Listeria seeligeri*
	Metal ABC transporter ATP-binding protein	FLVAIEKEDITP	*Li8- Listeria monocytogenes*
	Metal ABC transporter ATP-binding protein	QVTLVDKPLTYWR	*Li1- Listeria welshmeri*
	Zinc ABC transporter substrate-binding protein AdcA	KTNTSDKTADGK	*Li2- Listeria seeligeri*
	Putative high-affinity zinc ABC transporter (Zn(II)-binding lipoprotein)	LAGC[160.03]GANSNASGEK	*Li1- Listeria welshmeri*
	Pheromone ABC transporter	ALALAIDKQ	*Li9- Listeria ivanovi*
	Pheromone ABC transporter	KALALAIDKQ	*Li9- Listeria ivanovi*
	Thiol reductant ABC exporter subunit CydC	ELM*EAEPRYQR	*Li2- Listeria seeligeri*
Other transporters	Arsenic transporter	NAGLTDVLAR	*Li2- Listeria seeligeri*
	Arsenical pump-driving ATPase	AEDISGDALRD	*Li2- Listeria seeligeri*
	Cadmium, zinc and cobalt-transporting ATPase	VLIGGDIVKR	*Li8- Listeria monocytogenes*
	Chloramphenicol/florfenicol efflux MFS transporter	PPVLFNNK	*Li2- Listeria seeligeri*
	EmrB multiple drug resistance efflux pump MFS transporter	M*DKSKQEEDEEQV	*Li8- Listeria monocytogenes*
	Multidrug efflux SMR transporter	ILSGVVVLNLYGPGH	*Li3- Listeria innocua*
	Multidrug export protein mepA	DM*TKGNPTKLI	*Li3- Listeria innocua*

	Multidrug efflux MFS transporter	AALGM*AIAMILMGLV	*Li9- Listeria ivanovi*
	Multidrug efflux MFS transporter	AALGMAIAM*ILMGLV	*Li9- Listeria ivanovi*
	Multidrug efflux MFS transporter	PDFGVLMVAR	*Li3- Listeria innocua*
	RND superfamily drug exporter	LNDAIPVFAG	*Li2- Listeria seeligeri*
	Putrescine importer PuuP	QIIHSYPDGGGA	*Li4- Listeria monocytogenes*

M* methionine oxidation; C* carbamidomethylation of Cys.

Table S6. Peptides corresponding to other virulence factors, identified in the *Listeria* strains analyzed.

Function	Protein	Sequence	Strain
Other virulence factors	Integrase recombinase	QNKQPNSAIK	*Li1- Listeria welshmeri*
	Integrase	QAVFSYNYGFYNTKR	*Li2- Listeria seeligeri*
	Integrase	QFKEDIIMVAVG	*Li4- Listeria monocytogenes*
	Integrase	GDIDNKM*ER	*Li2- Listeria seeligeri*
	Integrase	M*LASGKYGQR	*Li9- Listeria ivanovi*
	Integrase	EGYRLLK	*Li4- Listeria monocytogenes*
	Integrase	GEALALRYENFNGSSM*VVDGTLDMGEK	*Li2- Listeria seeligeri*
	Integrase	FVVVAEEWFQ	*Li2- Listeria seeligeri*
	Integrase	KTRGENPPK	*Li3- Listeria innocua*
	Integrase	EKLIEELYR	*Li4- Listeria monocytogenes*
	Integrase	INDFLGNPK	*Li2- Listeria seeligeri*
	Integrase	LEKKFLDE	*Li4- Listeria monocytogenes*
	Integrase	ENEKSAAQKE	*Li2- Listeria seeligeri*
	Integrase	LLIDNGENITVIK	*Li2- Listeria seeligeri*
	Recombinase	RSVVM*NPR	*Li2- Listeria seeligeri*
	Recombinase	LNNLVVC*GK	*Li2- Listeria seeligeri*
	Recombinase	NITVIPAR	*Li7- Listeria monocytogenes*
	IS21 family transposase	QFAKDARFTPI	*Li4- Listeria monocytogenes*

	IS3 family transposase	LENEFGIIYSR	*Li7- Listeria monocytogenes*
	IS30 family transposase	ASKRNNIPR	*Li4- Listeria monocytogenes*
	Transposase B of Tn554	KVGNGINK	*Li2- Listeria seeligeri*
	Transposase B of Tn555	VGNGINKKEK	*Li2- Listeria seeligeri*
	Transposase	KKM*ANWLWEK	*Li4- Listeria monocytogenes*
	Tn3 family transposase	VPVGVSAIKADVNP	*Li4- Listeria monocytogenes*
	Tn3 family transposase	IDNLILNVSGN	*Li8- Listeria monocytogenes*
	Tn3 family transposase	M*AEATPGLTYKQLA	*Li2- Listeria seeligeri*
	Tn3 family transposase	SKENITQILR	*Li8- Listeria monocytogenes*
	IS200/IS605 family transposase	MSNDDKSLAHTRWN	*Li4- Listeria monocytogenes*
	IS30 family transposase	EILRSLDKPMSGNK	*Li4- Listeria monocytogenes*
	Transposase IS116/IS110/IS902 family protein	AYAGIDIRR	*Li3- Listeria innocua*
	Transposase	KIANMTSVLK	*Li4- Listeria monocytogenes*
	Transposase	EQKGFDGGKK	*Li4- Listeria monocytogenes*
	Transposase	KGIVGNEIAR	*Li8- Listeria monocytogenes*
	Mutator family transposase	FPNTDSAER	*Li2- Listeria seeligeri*
	Plasmid recombination enzyme	KDVETPEAVAAR	*Li2- Listeria seeligeri*
	Plasmid pRiA4b ORF-3 family protein	TEKEGGMPKLK	*Li4- Listeria monocytogenes*
	Plasmid pRiA4b ORF-3 family protein	EGGM*PKL	*Li6- Listeria monocytogenes*
	Plasmid mobilization relaxosome protein MobC	LSEEQYNKLK	*Li7- Listeria monocytogenes*
	Bacillus transposase protein	GISAAADNIK	*Li2- Listeria seeligeri*
	Phage infection protein, YhgE	ATIASNLVK	*Li4- Listeria monocytogenes*
	Phage infection protein, YhgE	ETIVNGINR	*Li4- Listeria monocytogenes*
	Phage infection protein, YhgE	LNDGVSTLAAG	*Li2- Listeria seeligeri*

	Phage infection protein, YhgE	SQLQSGMAQLSAGSAKLEQGLR	*Li3- Listeria innocua*

M* methionine oxidation; C* carbamidomethylation of Cys.
